# Looking beyond the hippocampus: old and new neurological targets for understanding memory disorders

**DOI:** 10.1098/rspb.2014.0565

**Published:** 2014-07-07

**Authors:** John P. Aggleton

**Affiliations:** School of Psychology, Cardiff University, Park Place, Cardiff, Wales CF10 3AT, UK

**Keywords:** amnesia, dementia, mamillary bodies, memory, retrosplenial cortex, thalamus

## Abstract

Although anterograde amnesia can occur after damage in various brain sites, hippocampal dysfunction is usually seen as the ultimate cause of the failure to learn new episodic information. This assumption is supported by anatomical evidence showing direct hippocampal connections with all other sites implicated in causing anterograde amnesia. Likewise, behavioural and clinical evidence would seem to strengthen the established notion of an episodic memory system emanating from the hippocampus. There is, however, growing evidence that key, interconnected sites may also regulate the hippocampus, reflecting a more balanced, integrated network that enables learning. Recent behavioural evidence strongly suggests that medial diencephalic structures have some mnemonic functions independent of the hippocampus, which can then act upon the hippocampus. Anatomical findings now reveal that nucleus reuniens and the retrosplenial cortex provide parallel, disynaptic routes for prefrontal control of hippocampal activity. There is also growing clinical evidence that retrosplenial cortex dysfunctions contribute to both anterograde amnesia and the earliest stages of Alzheimer's disease, revealing the potential significance of this area for clinical studies. This array of findings underlines the importance of redressing the balance and the value of looking beyond the hippocampus when seeking to explain failures in learning new episodic information.

## 1. A tragic start

On 13 June 1886, the eminent psychiatrist Bernhard von Gudden walked with Ludwig II, King of Bavaria, by the side of Lake Starnberg near Munich. Neither was to return. Both Bernhard von Gudden and Ludwig, also known as ‘the dream king’, were found floating in the lake. Whether it was murder, an accident or suicide remains a mystery [1]. Gudden was a brilliant anatomist who, before his untimely death, had described in new levels of detail the pathways linking the hippocampus to the mamillary bodies [2], a structure within the medial diencephalon. Just 10 years after Gudden's untimely death, the first functional implications of these anatomical findings were uncovered by his son, Hans Gudden [3].

In 1896, Hans Gudden described pathological changes in the brains of alcoholics [3], some of whom suffered from Korsakoff's syndrome [4]. In this amnesic condition, there is both a failure to remember events from before the onset of the amnesia (retrograde amnesia) and a failure to learn new events after the onset of the amnesia (anterograde amnesia). Gudden reported that the mamillary bodies were atrophied in those alcoholic cases with probable Korsakoff's syndrome, a discovery subsequently confirmed in numerous neuropathological studies [5–7]. It has also emerged that Korsakoff's syndrome is almost always accompanied by other medial diencephalic pathologies, leading to much debate over the causes of the amnesia [5–7]. Current evidence now favours Hans Gudden's original suspicion, with the pathway from the mamillary bodies → mamillothalamic tract → anterior thalamic nuclei seen as a leading cause of the anterograde amnesia [7–9].

Despite growing numbers of studies on Korsakoff's syndrome in the first half of the twentieth century, the focus gradually turned to the hippocampus and its adjacent cortical areas (the parahippocampal region). The Russian neurologist Bekhterev [10] is often credited as the first person (in 1900) to appreciate the involvement of the hippocampus in memory. Bekhterev's research was, however, suppressed after his death, quite possibly on the direct orders of Stalin [11]. Indeed, there are grounds to suppose that Stalin himself ordered Bekhterev's death following unguarded remarks concerning the dictator [11].

The study that truly pushed the hippocampus into the forefront of memory research was the description of the amnesic patient H.M. [12,13]. The amnesic H.M., or Henry Mollaison as we discovered after his death in 2008, is undoubtedly the most famous single-case in neuropsychology. In 1953, the surgeon William Scoville bilaterally removed tissue in the medial temporal lobes of H.M.'s brain in an attempt to treat his epilepsy [12]. The descriptions of his profound and permanent anterograde amnesia, which contrasted with his preserved short-term memory, semantic knowledge and IQ, were groundbreaking in the way they helped to establish divisions across cognitive domains [12,14]. Ironically, H.M. always remained unaware of his fame.

Studies on the patient H.M. are also often credited as providing the first convincing evidence that the hippocampus is vital for new episodic memory. The story is in reality more complex and even more tragic. Henry Mollaison was, in fact, one of a group of nine patients that received bilateral removals of medial temporal lobe tissue [12]. The procedure in the other eight patients was intended to relieve psychiatric problems. Subsequent group comparisons based on the amount of bilateral hippocampal tissue thought to have been removed led to the conclusion that the loss of this structure was especially associated with memory failure ([13], but see [15]). Sadly, the experimental surgeries failed to resolve the patients’ psychiatric problems. Not surprisingly, such procedures ceased, leaving H.M. unique.

Despite Hans Gudden's lead, which initially directed attention to the mamillary bodies, it is the hippocampus that dominates research into memory loss and memory formation. Although the groundbreaking clinical study involving H.M. did not, in fact, provide definitive evidence for the importance of the hippocampus [15], the intervening years have repeatedly strengthened the view that this structure is vital for learning episodic information [16,17]. As a consequence, researchers have long held a hippocampal-centred view of long-term memory. There are, for example, approximately 30 published papers linking the hippocampus with memory for every one paper that links medial diencephalic sites with memory (ISI, Web of Science).

In this Perspective, anatomical, behavioural and clinical findings are integrated to highlight the importance of sites beyond the medial temporal lobe for episodic memory. It is argued that these extra-hippocampal sites should be carefully considered when trying to understand how diseases, including dementias, can have such devastating effects on memory. It is also argued that many of these sites have important roles for hippocampal function, rather than primarily acting as a downstream relay from the hippocampus. Hereafter in this review, the term hippocampus refers to the CA fields and the dentate gyrus, whereas the term hippocampal formation additionally includes the subicular cortices.

## 2. Anatomical insights

Clinical studies have shown that more than one brain region is required for normal long-term memory. As a consequence, a critical anatomical question is whether the hippocampal formation is connected to these other sites repeatedly implicated in anterograde amnesia [18–20]. Among these other sites, the mamillary bodies and anterior thalamic nuclei have already been mentioned, but additional sites linked with anterograde amnesia include the retrosplenial cortex within the cingulate region [21–23], the laterodorsal thalamic nucleus [21] and nuclei in the basal forebrain, a region that includes the septum and diagonal band [21,24].

**Figure 1.**
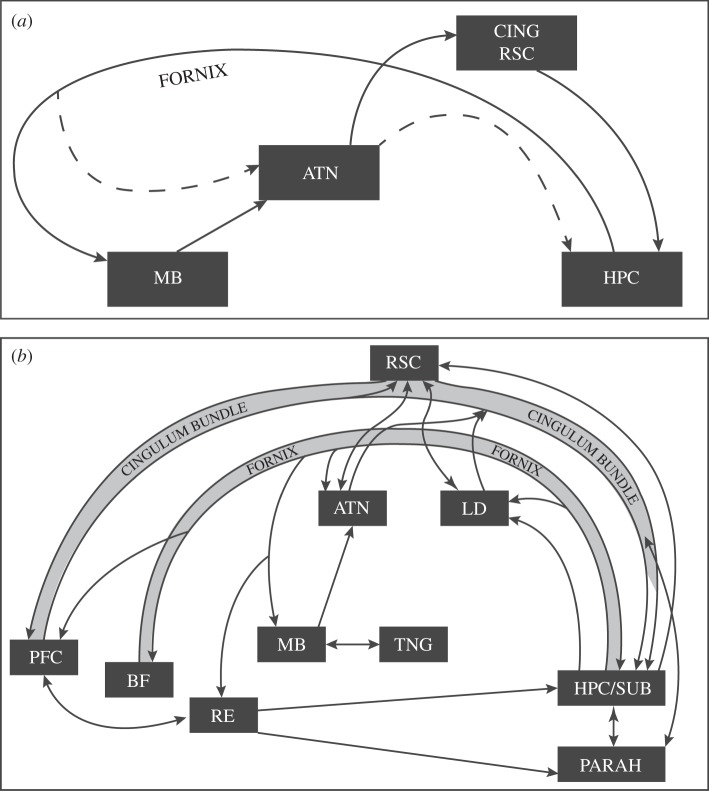
Interconnections between sites implicated in anterograde amnesia. (*a*) Depiction of the connections (solid lines) that comprise the Papez circuit [25], upon which the Delay and Brion memory circuit [5] was placed, with additional connections from the ‘extended hippocampal system’ [26] shown by dashed lines. (*b*) A more extensive (though still incomplete) depiction of the direct connections between cores sites implicated in episodic memory. ATN, anterior thalamic nuclei; BF, basal forebrain (including septum and diagonal band); CING, cingulate cortex (including retrosplenial cortex); HPC/SUB, hippocampal formation (including subiculum); LD, laterodorsal thalamic nucleus; MB, mamillary bodies; PARAH, parahippocampal region; PFC, prefrontal cortex; RE, nucleus reuniens of the thalamus; RSC, retrosplenial cortex; TNG, tegmental nucleus of Gudden.

All of these structures linked with amnesia receive direct inputs from the hippocampal formation (figure 1), many of which arise from the subicular cortices [18,20]. These same structures also project directly back upon the hippocampal formation, with the exception of the mamillary bodies (figure 1). In the case of the mamillary bodies, the majority of its projections pass via the mamillothalamic tract to the anterior thalamic nuclei. These thalamic nuclei are densely and reciprocally interconnected with the retrosplenial cortex. In addition, both the anterior thalamic nuclei and the retrosplenial cortex project to the hippocampal formation, terminating in the subicular cortices [27,28]. In this way, the hippocampal formation, mamillary bodies, anterior thalamic nuclei and retrosplenial cortex form a serial network (figure 1*a*), initially referred to as Papez’ circuit [25], but subsequently called the Delay and Brion circuit [5] when it is more explicitly linked to memory and amnesia. These sites are thought to form an interconnected system for episodic memory under the principal control of the hippocampal formation [5,25]. This notion is emphasized by use of the term circuit, which implies a return back to the start point (figure 1*a*).

Two additional points emerge from this brief consideration of anatomy. The first concerns the strategic status of the fornix. This tract connects the hippocampal formation with other sites linked to amnesia, the retrosplenial cortex being the sole exception ([19,20]; figure 1). For this reason, the consequences of fornix damage on memory should prove highly informative (see §4). The second point concerns the reciprocal hippocampal connections with the basal forebrain, which includes the septum and diagonal band. These latter nuclei are not closely connected with the mamillary bodies or the anterior thalamic nuclei, suggesting that they might represent a quite different type of hippocampal interaction (figure 1*b*). One difference is that these basal forebrain nuclei, which are the principal sources of the extrinsic cholinergic projections to the hippocampal formation, have diffuse termination sites that reach across the structure [18,29]. By contrast, the projections from the anterior thalamic nuclei are confined to the subicular cortices [28]. The implication is that these basal forebrain inputs have broad modulatory roles, lacking the fine resolution associated with high information throughput. By contrast, the hippocampal connections with the medial diencephalon appear to be better designed for information transfer. There is, for example, segregation rather than convergence in the organization of the projections from the hippocampal formation to the anterior thalamus and mamillary bodies [30,31]. The sources of these projections from within the subiculum are separated by laminar, as well as by their location along the proximal—distal axis of the subiculum [31,32]. Further segregation is found in the projections from the mamillary bodies to the anterior thalamic nuclei [33]. The large numbers of fibres in the projections from the hippocampal formation to the mamillary bodies, and from there to the anterior thalamus [34] are again consistent with a role in information transfer, despite the likely compaction of hippocampal inputs within the medial diencephalon.

A further issue concerns the interactions between the hippocampal formation and prefrontal cortex, assumed to be important for the cognitive control of mnemonic processes. While projections from the hippocampal formation (from CA1 and the subiculum) terminate in parts of the medial and orbital frontal prefrontal cortex [20], reciprocal connections to the hippocampal formation appear very limited [20,35]. Consequently, there is particular interest in the discovery of two, dysnaptic routes from prefrontal cortex to the hippocampus [36]. One route is via the retrosplenial cortex, the other route is via nucleus reuniens of the thalamus. Nucleus reuniens, which is located ventral to the anterior thalamic nuclei, has very dense projections that terminate in CA1, as well as the subiculum [36]. Thus, based on their patterns of termination, it can be seen that the three subcortical sites under consideration (the basal forebrain, the anterior thalamic nuclei and nucleus reuniens) have different types of interactions with the hippocampal formation, suggesting different roles.

## 3. Behavioural insights

The cell loss in amnesia very rarely respects neuroanatomical boundaries (see §4). One solution has been to examine the impact of highly selective brain lesions in other animals. At the same time, there are drawbacks. To test human episodic memory, participants are typically required to recall information such as word lists or abstract designs. This recall is often assumed to require an active, introspective search through past time [37]. Unfortunately, we have no way of testing for this same introspective process in animals. While it had been supposed that recognition memory could be used to model amnesia, as it is often impaired in amnesia and can readily be tested in both human and animal subjects using comparable methods [17], this approach has been challenged. There are, for example, amnesics who show a disproportionate sparing of recognition when compared with their loss of recall [38–40].

An alternative approach is to devise behavioural tasks that capture key aspects of episodic memory, without having to assume the presence of active recall. Such tasks typically involve demonstrating that the animal has simultaneously learnt the what? where? and when? of a singular event [41–43]. Studies using these complex problems in rodents have demonstrated the importance of the hippocampal formation and fornix for ‘episodic-like’ memory, though cholinergic lesions of the medial septum and diagonal band can spare performance [43–45]. One obvious limitation is that a deficit in just one of the three elements (what? where? and when?) may be sufficient to impair the entire task, akin to a domino effect (but see [45]).

A different strategy is to explore tasks more specific for spatial or temporal memory. Disconnection methods have shown that the hippocampal formation requires the anterior thalamic nuclei and the retrosplenial cortex to support spatial learning [46–48]. Likewise, the anterior thalamic nuclei work in close conjunction with the retrosplenial cortex [46]. By combining lesions, it has also been possible to show how the fornix and the mamillary bodies are both similarly needed for object-in-place learning by monkeys [49], a task thought to tax aspects of episodic memory. While these behavioural findings establish functional links in distributed systems, they say nothing about the direction of importance. The prevailing assumption has been that these other sites are primarily controlled by the hippocampus [5,35]. This view has been challenged.

One challenge came from comparisons between the functions of structures within the medial temporal lobe, where the dominant view had placed the hippocampal formation on the top of a functional and anatomical hierarchy [17]. There is now considerable evidence that the perirhinal cortex can support recognition memory, independent of the hippocampus [20,35]. In particular, experiments show that the perirhinal cortex can provide a familiarity signal to help differentiate novel from repeated stimuli [35,50–52].

Initial evidence that the mamillary bodies and anterior thalamic nuclei could also provide new memory-related information, which would then impact upon the hippocampal formation, was first signalled by research on head direction cells. These cells act like a compass, providing information about the direction an animal is facing [53]. Head direction cells occur within brain structures strongly linked to memory and amnesia [54], thus they are found within parts of the mamillary bodies, anterior thalamic nuclei, laterodorsal thalamic nucleus, retrosplenial cortex and postsubiculum (presubiculum) of the hippocampal formation [53]. Furthermore, the head direction signals in the lateral mamillary nucleus and anterodorsal thalamic nuclei are upstream of the hippocampal formation, such that the loss of these diencephalic sites can abolish the hippocampal head direction signal [55–57]. While lesions of the mamillary bodies do not appear to affect hippocampal place cells [57], anterior thalamic lesions degrade the spatial coherence and information content from hippocampal place fields [58]. Consequently, the integrity of the anterior thalamic nuclei is required for broad aspects of hippocampus spatial processing.

The next evidence takes us back to Bernhard von Gudden. The medial mamillary bodies are densely interconnected with the ventral tegmental nucleus of Gudden, but these tegmental nuclei do not receive inputs from the hippocampal formation. Thus, the finding by Vann [59,60] that lesions in both the ventral tegmental nucleus and the mamillary bodies disrupt the same spatial learning tasks, which are also sensitive to hippocampal damage, suggests further upstream influences. Stronger support for this view comes from the surprising finding that selectively disconnecting the fornical projections to the mamillary bodies has limited effects on standard spatial learning tasks [60,61]. Critically, lesions of the mamillothalamic tract and ventral tegmental nucleus of Gudden are significantly more disruptive to tests of spatial memory than postcommissural fornix lesions, which selectively disconnect the hippocampal → mamillary body inputs [62]. These findings by Vann [59,60] challenge the standard serial system view, where hippocampal inputs drive mamillary body function ([26]; figure 1*a*). While it might be supposed that this sparing occurs because the direct projections from the hippocampal formation to the anterior thalamic nuclei can compensate for the loss of the hippocampal inputs to the mamillary bodies (figure 1), this account will not explain why the same spatial tasks are appreciably more sensitive to lesions of the mamillary bodies or the mamillothalamic tract [59,60]. These results are, therefore, inconsistent with the standard hippocampal-centred view as they reveal the significance of non-hippocampal inputs into this system.

It is important to appreciate that these lesion findings do not merely reflect a loss of head direction information. This possibility can be excluded because the ventral tegmental nucleus of Gudden is not part of the head direction system. Furthermore, lesions of the mamillary bodies and anterior thalamic nuclei that target the head direction areas within these structures have relatively mild effects on spatial learning when compared with more complete lesions of these same structures [54,60,63–65].

An additional way in which the anterior thalamic nuclei may regulate hippocampal activity has emerged from studies of neurogenesis. High-frequency stimulation in the anterior thalamic nuclei can increase neurogenesis in the dentate gyrus of the rodent hippocampus [66,67] and aid the performance of memory tasks [68]. Furthermore, pharmacological lesions of the anterior thalamic nuclei suppress hippocampal neurogenesis [69]. These findings are particularly intriguing given the considerable evidence that hippocampal neurogenesis has an important role in learning and memory [70].

Finally, based on its prefrontal connectivity, it would seem that nucleus reuniens is particularly well placed to moderate hippocampal activity [36]. Consistent with this view, lesions of nucleus reuniens can decrease behavioural flexibility and disrupt strategy learning [71–73]. Other lesion effects include deficits in radial-arm maze performance [74,75], slower water-maze location learning [74] and a disruption of long-term spatial consolidation [75]. A particular role in processing the information from the prefrontal cortex to the hippocampus that regulates context specificity has also been discovered [76]. These studies reveal the emerging importance of nucleus reuniens for hippocampal processing [72], though with functions different from those attributed to the anterior thalamic nuclei [77].

## 4. Clinical insights

As noted in the Introduction, the mamillary bodies were first implicated in Korsakoff's syndrome by Hans Gudden in 1896. However, from just studying Korsakoff's syndrome, it has not been possible to determine whether mamillary body damage is sufficient to induce amnesia. Other clinical information has since strengthened the argument that these diencephalic nuclei are closely involved in learning and memory [8].

One remarkable set of findings comes from two unfortunate patients who incurred traumatic injuries to the base of the brain after an object was forced up their nose. In the case of B.J. [78] the object was a billiard cue, in the case of N.A. [79] the object was a miniature fencing foil. Patient B.J. suffered selective bilateral damage to his mamillary bodies, resulting in a relatively mild, anterograde amnesia [78,80]. His recognition memory appeared to be largely spared. Patient N.A. suffered unilateral diencephalic damage that included his left mamillothalamic tract, resulting in a loss of verbal long-term memory. In both cases, the principal deficit was a loss of episodic memory. These findings concur with an extensive review of thalamic strokes [9], which found that damage to the mamillothalamic tract was the best predictor of memory loss.

Further evidence comes from studies of patients with colloid cysts in the third ventricle. These cysts often adhere to the fornix and their removal is sometimes associated with damage to this tract. Severance of the fornix, which will disconnect the hippocampal formation from both the medial diencephalon and basal forebrain, is consistently associated with amnesia [81,82]. In one of the largest studies of colloid cyst patients, volumetric measures were taken in a range of medial temporal and diencephalic sites [38]. The degree of mamillary body atrophy consistently correlated with the ability to recall episodic memory [38]. By contrast, recognition memory seemed largely unaffected by mamillary atrophy [38]. Further tests showed that the relatively intact recognition memory performance was due to a sparing of familiarity-based recognition, while recollective-based recognition remained linked to the degree of mamillary body atrophy [83]. The implication is that the mamillary bodies are required for the normal recall of episodic information, while the familiarity signal, which can support recognition memory, is dependent on other structures. This finding is, in turn, closely linked to parallel evidence that the hippocampus is particularly important for recollective-based, but not familiarity, recognition [26,51].

It is possible to use diffusion tensor imaging (DTI) to quantify the status of specific white matter tracts. Using this approach, it has been shown that fornix integrity correlates with episodic memory in the normal population, though not with familiarity-based recognition [84]. This dissociation reflects that found in the colloid cyst patients with atrophied mamillary bodies [38,83]. Using a similar DTI-based methodology, this same relationship between fornix status and memory is found in the elderly, though this correlation breaks down in matched elderly patients with mild cognitive impairment [85,86]. These imaging studies not only show how vulnerable the fornix is in this disorder but also provide intriguing evidence that there is a redistribution of cognitive function away from the fornix to less compromised pathways [86]. Mild cognitive impairment, which is often a prodromal stage of Alzheimer's disease, is of particular interest as the loss of episodic memory is typically the most evident symptom. Patients with behavioural variant frontotemporal dementia can also show severe deficits in episodic memory, which in this condition has been linked with the degree of atrophy in both the fornix and anterior thalamic nuclei [80], further underlining the significance of structures in the Papez circuit beyond the hippocampus (figure 1).

Studies of Alzheimer's disease also increasingly point to the significance of pathology beyond the temporal lobes. Two classic, diagnostic pathological features are amyloid plaques and neurofibrillary tangles. Descriptions of the time course of Alzheimer's disease, based on the accumulation of tangles, reveal that the first abnormalities (Stage I) are typically seen in the parahippocampal region [87,88]. By Stage III, when cognitive symptoms first become evident, tangles appear in the hippocampal formation [87,88]. Not surprisingly, such information has reinforced the focus on the hippocampal formation when trying to understand memory loss in Alzheimer's disease. Although tangles and plaques also appear in the anterior thalamic nuclei at similar stages to the hippocampus [87,89], these thalamic pathologies have often been interpreted as a part of a cascade of downstream events that follow the projections from the hippocampal formation [89]. This interpretation is challenged by imaging studies of plaque deposition [90] and metabolic activity [91,92]. The earliest, consistent decreases in metabolic activity during the progression of Alzheimer's disease are found not in the hippocampus, but in the retrosplenial cortex and adjacent parts of the posterior cingulate cortex [91,92]. Subsequent studies of mild cognitive impairment have demonstrated that the retrosplenial cortex is the earliest brain site to show consistent activity loss [93]. These findings prompted neuropathologists to look again at the retrosplenial cortex in the post-mortem brains of Alzheimer's disease patients. These studies found cell loss consistent with a much earlier involvement in the disease than previously assumed [94–96], including in those patients who convert from mild cognitive impairment to Alzheimer's disease [97]. Indeed, thinning in the posterior cingulate cortex could be identified in cases of familial Alzheimer's disease up to 1.8 years prior to diagnosis, with even earlier changes in the adjacent precuneus cortex ([98]; see also [90]).

Such findings suggest that studies on the retrosplenial cortex, as well as the hippocampus, may prove highly informative when tracing the early progression of this dementia. By association, the anterior thalamic nuclei are also implicated; given their dense, reciprocal connections with both structures [23,77]. It is, therefore, of note that pre-symptomatic familial cases of Alzheimer's disease show increased amyloid load in the thalamus [99] as well as evidence of thalamic atrophy, which was detected on average 5.6 years prior to expected symptom onset [100]. These results complement the reports of thalamic atrophy in mild cognitive impairment [97]. The emergence of this more distributed view of early Alzheimer pathology accompanies the current notion that this disease has a long incubation period, with a prodromal phase that may stretch back many decades [101]. This conceptual approach has led to the search for possible biomarkers of the earliest stages of the disorder, i.e. at those stages when an intervention may be most beneficial. Functional magnetic resonance imaging (fMRI) studies of people at high risk for Alzheimer's disease [101] have found abnormalities in resting state connectivity in the posterior cingulate area, including the retrosplenial cortex, along with the hippocampal formation [101,102]. These abnormalities include changes in the ‘default mode network’ [23,101]. These findings underline the need to understand the pathology of Alzheimer's disease in its broadest anatomical sense.

## 5. Conclusion

The idea that multiple brain sites support episodic memory is far from new. Indeed, up until the last 15 years, it was popular to assume that medial temporal lobe amnesia and medial diencephalic amnesia were distinct syndromes with independent causes. More recent research has consistently shown how the similarities between these conditions far outweigh the differences, leading to a more unified view [21,103]. These neuropsychological findings further encouraged the idea that the hippocampus should be at the centre of memory-related research. In many instances, this focus is entirely appropriate, yet it may also be misleading.

One issue, initially highlighted by studies of hypoxia, is that of hidden, or covert, pathology. The lack of oxygen to the brain is a cause of anterograde amnesia, which is consistently associated with hippocampal cell loss. A debate emerged, however, over whether hippocampal atrophy could fully explain the pattern of cognitive loss in those clinical and experimental hypoxic cases with seemingly selective pathology [104,105]. In many instances, it appeared that the cognitive deficits were greater than those that could be atributed to the overt pathology [104,106], a view supported by volumetric imaging studies of hypoxia, which reported diffuse changes that would be hard to detect using stardard post-mortem techniques.

In a second form of covert pathology, damage to structure A brings about subtle changes to structure B that render structure B highly dysfunctional, even though its appearance as judged by standard histological measures seems intact. One source of evidence comes from functional imaging studies, which have shown how both medial temporal lobe amnesias and medial diencephalic amnesias are associated with hypoactivity in the posterior cingulate region, centred in the retrosplenial cortex [107,108]. The retrosplenial cortex has dense reciprocal connections with both regions [23], suggesting that this hypoactivity is a secondary consequence of the primary pathologies. Experimental studies in rodents, which have sought to test this possibility more directly, have found that lesions in the mamillothalamic tract, anterior thalamic nuclei and hippocampal formation all bring about striking molecular changes in the retrosplenial cortex, indicating that this cortical area is especially vulnerable to distal damage. The nature of these secondary lesion effects strongly suggests a disruption of retrosplenial plasticity [103,109–112], along with a reduction in metabolic activity [113,114]. Obvious implications can be seen for the retrosplenial cortex hypoactivity found in the earliest stages of Alzheimer's disease [91–93]. Despite these activity changes, the structural appearance of the retrosplenial cortex after anterior thalamic lesions in rodents is almost unaffected. Furthermore, such distal lesion effects are not confined to the retrosplenial cortex as anterior thalamic lesions also disrupt plasticity-related mechanisms in the hippocampal formation [115–117]. This array of covert limbic changes could exacerbate the impact of the primary pathology on memory, whether it was in the medial diencephalon or the medial temporal lobe.

The purpose of this Perspective is not to argue that the hippocampus should be ignored as a target for research into memory and memory disorders. Clearly, it is crucial that this structure is studied intensively. At the same time, researchers need to appreciate that it is just one of a number of potential target sites. There is the genuine possibility of error when, for example, animal researchers assume that a behavioural phenotype involving deficits in spatial learning must stem from hippocampal pathology. This need not be the case. Furthermore, even if hippocampal abnormalities are found, they may be secondary to the site that is principally responsible for the spatial deficits. It might be supposed that fMRI studies of memory would ensure that such anatomical attribution errors would stop. While fMRI studies have revealed numerous sites, e.g. within prefrontal cortex, that are activated during memory encoding and retrieval, these same sites do not appear to be vital for memory when analysed using classic neuropsychological methods [35,51,118]. The problem, therefore, remains in deciding where to focus research efforts, with the hippocampus seemingly the default option. Just as Bernhard von Gudden did 135 years ago, we should remember to look beyond the hippocampus.

## Funding statement

The author would like to thank the MRC, Wellcome Trust and a Royal Society Wolfson Research Merit Award for funding his research.
